# Common practical questions – and answers – at the British Association for Psychopharmacology child and adolescent psychopharmacology course

**DOI:** 10.1177/02698811221140005

**Published:** 2022-12-07

**Authors:** Samuele Cortese, Frank MC Besag, Bruce Clark, Chris Hollis, Joseph Kilgariff, Carmen Moreno, Dasha Nicholls, Paul Wilkinson, Marc Woodbury-Smith, Aditya Sharma

**Affiliations:** 1 Centre for Innovation in Mental Health, School of Psychology, Faculty of Environmental and Life Sciences, University of Southampton, Southampton, UK; 2 Solent NHS Trust, Southampton, UK; 3 Hassenfeld Children’s Hospital at NYU Langone, New York University Child Study Center, New York City, NY, USA; 4 Mental Health and Clinical Neurosciences, School of Medicine, University of Nottingham, Nottingham, UK; 5 UCL School of Pharmacy, London, UK; 6 East London Foundation NHS Trust, Bedfordshire, UK; 7 Institute of Psychiatry, Psychology and Neuroscience, KCL, London, UK; 8 National Specialist Clinic for Young People with OCD, BDD and Related Disorders, South London and Maudsley NHS Foundation Trust, London, UK; 9 Department of Child and Adolescent Psychiatry, Nottinghamshire Healthcare NHS Foundation Trust, Queen’s Medical Centre, Nottingham, UK; 10 National Institute of Mental Health (NIHR) MindTech Medtech Co-operative, Institute of Mental Health, Nottingham, UK; 11 NIHR Nottingham Biomedical Research Centre, Mental Health & Technology Theme, Institute of Mental Health, Nottingham, UK; 12 Department of Child and Adolescent Psychiatry, Institute of Psychiatry and Mental Health, Hospital General Universitario Gregorio Marañón, IiSGM, CIBERSAM, ISCIII, School of Medicine, Universidad Complutense, Madrid, Spain; 13 Division of Psychiatry, Imperial College London, London, UK; 14 NIHR ARC Northwest, London, UK; 15 School of Clinical Medicine, University of Cambridge, Cambridge, Cambridgeshire, UK; 16 Cambridgeshire and Peterborough NHS Foundation Trust, Cambridgeshire, UK; 17 Biosciences Institute, Newcastle University, Newcastle upon Tyne, UK; 18 Academic Psychiatry, Translational and Clinical Research Institute, Newcastle University, Newcastle upon Tyne, UK; 19 Specialist Adolescent Mood Disorders Service (SAMS), Cumbria, Northumberland, Tyne and Wear NHS Foundation Trust, Newcastle upon Tyne, UK

**Keywords:** Psychopharmacology, child, adolescents, young people, evidence, expert opinion

## Abstract

The British Association for Psychopharmacology course on child and adolescent psychopharmacology has been run for more than 20 years and is currently a very popular course, attracting around 140 delegates/year from across the United Kingdom and abroad. As Faculty of recent sessions of the course, we have selected the most common questions we have been asked in recent years and provided evidence-based and/or expert-informed answers. We have included 27 questions and answers related to attention-deficit/hyperactivity disorder, anxiety and depressive disorders, autism spectrum disorder, bipolar disorder, eating disorders, epilepsy (in differential diagnosis or comorbid with mental health conditions), obsessive-compulsive disorder, personality disorders, psychotic spectrum disorders, and tics/Tourette syndrome in children and young people. We hope that this article will be helpful for prescribers in their daily clinical practice and we look forward to further, high-level evidence informing the answers to these and other questions in child and adolescent psychopharmacology.

## Introduction

Since 2001, the British Association for Psychopharmacology (BAP) has run a course (‘clinical certificate’) on child and adolescent psychopharmacology, that has become more and more popular over the years and has attracted prescribers (around 140/year) from across the United Kingdom and abroad. As Faculty of the recent sessions of the course, we have reported here the most frequent practical questions that we have been asked by the delegates, and our answers. In general, the questions were related to aspects/topics for which there is not a solid evidence base yet. Therefore, our answers reflect the available evidence alongside our clinical expertise and expert opinion. We have grouped the questions and answers by disorder to which they refer, in alphabetical order. In writing this article we have adhered to the Neuroscience-based Nomenclature (NbN), a version of which (NbN C&A) is available also for child and adolescent psychopharmacology (Cortese et al., 2022). The NbN (C&A) prompts researchers and clinicians to label psychotropic medications based on their putative psychopharmacological mechanisms of action, rather than referring to their putative indications (e.g. *antidepressants* or *antipsychotics*), which may be misleading. Accordingly, we have indicated the mode of action when we first mention a medication. For ease of reading, we have however used the former, traditional terminology in the remaining parts of the text.

Table 1 summarises all the questions and answers.

**Table 1.** Summary of questions and answers grouped by disorder (in alphabetical order).

**ADHD** **1. Can I use doses of psychostimulants beyond the maximum licensed or recommended ones?** Doses beyond those recommended in guidelines should not be used routinely, but could be considered in selected cases, particularly in patients with overweight when a partial response was obtained at the licensed dose. **2. What shall I do if a patient does not respond to psychostimulants?** Before considering non psychostimulants or polypharmacotherapy, check if: (a) you have titrated properly, (b) you have optimised the coverage during the day as needed, (c) you have targeted the right symptoms, (d) there is possible tolerance, (e) psychosocial factors/life events or any comorbidity could account for the symptomatology and (f) the diagnosis is correct **3. Does the concomitant use of cannabis impact on the effectiveness and tolerability of psychostimulants?** There is no evidence showing that, when combined with cannabis, psychostimulants are less well tolerated, but they might be less effective **ANXIETY AND DEPRESSIVE DISORDERS** **4. What do I do if a young person has depression and has not responded to two SSRIs and CBT?** After revising diagnosis/formulation and checking adherence, off-label options include mirtazapine or venlafaxine (the latter should be used cautiously due to possible associated increased suicidal ideation), and augmenting agents such as lamotrigine, antipsychotic, lithium or l-thyroxine. **5. If a patient has severe side effects (such as suicidality) on one SSRI, is it safe to try a second SSRI?** There is no robust evidence either way around the safety of this. If sertraline caused dangerous side effects, it would be better to try the more different fluoxetine than citalopram as second line **6. I see an adolescent with an anxiety disorder who a GP has started on propranolol. What should I do?** Stop it as there is no evidence it is effective (with the possible exception for children with ASD and anxiety) – it has significant side effects and it can be dangerous in overdose. **Autism Spectrum Disorder (ASD)** **7. Can I use SSRI medication to manage repetitive behaviours in a child with a diagnosis of ASD?** Overall, there is no meta-analytic evidence supporting SSRIs but it may be reasonable to consider an SSRI when other non-pharmacological treatment options have failed, given data from some individual studies. If SSRIs are inefficacious or poorly tolerated, risperidone, guanfacine or buspirone may be considered as an alternative. **8. What are my options in the management of aggression in a child with ASD?** This should be guided by the diagnosis/formulation. Risperidone or aripiprazole may be considered as pharmacological options. **9. What are my pharmacological choices for the management of aggression in a child with ASD when standard options seem to have failed?** Quetiapine, olanzapine, benzodiazepines (such as lorazepam, diazepam or clonazepam) or the combination of an antipsychotic with a benzodiazepine may all be considered, for the short term. **IPOLAR DISORDER (BD)** **10. How does one distinguish ADHD from BD?** In ADHD the symptoms are pervasive and generally chronic over time (with some variation in severity), while BD presents with an episodic course of symptoms. Importantly, ADHD is a disorder of the modulation of attention, motor activity and impulsivity, whereas BD is a disorder of mood. **11. How does one distinguish Borderline Personality Disorder (BPD)?** BD is a disorder of mood characterised by discrete mood episodes. BPD is characterised by a long-term pattern of unstable interpersonal relationships, distorted sense of self and marked affective instability. **12. How does one distinguish treatment-emergent affective switch from behavioural activation when using antidepressants and what is the management?** Even though they might lie on a continuum, manic spectrum symptoms involve a change in mood and behaviour with coexisting symptoms of grandiosity and euphoria, which are not found in the SSRI-induced activation syndrome. **EATING DISORDERS** **13. What additional considerations are needed when prescribing in young people with eating disorders?** An ECG should be performed before prescribing psychotropic medications given the cardiac risks secondary to malnutrition, and electrolyte abnormalities secondary to malnutrition or purging should be considered. In relation to anxiety about, and sensitivity to, side effects, it is important to clarify with the patient that the primary purpose of prescribing antipsychotic medication in patients with anorexia nervosa is to reduce overwhelming arousal during meal times and not directly to cause weight gain. **14. Should comorbidities in young people with eating disorders be treated separately?** Eating disorders can mimic or accentuate comorbidities; effective psychological treatments for eating disorders can result in improvements in other areas, and some pharmacological interventions are ineffective during the acute phase. However, if the history clearly suggests that untreated or undiagnosed mental disorder have preceded the onset of the eating disorder and a patient is not responding to competently delivered treatment as expected, treatment of comorbidity should be considered. **15. How might treatment approaches differ in young people with an eating disorder in the context of neurodevelopmental disorder?** In individuals with Binge Eating Disorder (BED), treatment of comorbid ADHD may be helpful in addressing difficulties in impulsivity and self-regulation that also characterise binge eating disorder (Lisdexamfetamine is approved in the United States by the FDA for BED in adults but it is not licenced in the United Kingdom for this indication). Treatment adaptations for comorbid ASD are in development. **EPILEPSY (in differential diagnosis or comorbid with mental conditions)** **16. What is the role of cannabidiol in the child with epilepsy and behavioural problems?** There are now sufficient high-quality studies to confirm the efficacy of cannabidiol in treating seizures in both adults and children. There is currently conflicting evidence with regard to either beneficial or adverse behavioural effects, although some suggestion of benefit is there. **17. If a child is having nocturnal episodes that are disturbing sleep, when should we be assessing for epilepsy as a possible cause?** Nocturnal frontal lobe seizures typically present with brief episodes that can occur multiple times in a single night, often with non-rhythmical movements. They are frequently accompanied by vocalisation and, in contrast to the situation for most other focal-onset seizures, it is often possible for the individual to recall the seizures afterwards. Nocturnal tonic (stiffening) seizures, generally with background history of epilepsy, may be difficult to distinguish from normal nocturnal movements but video EEG monitoring can be diagnostic. Finally, any child who loses skills for no apparent reason deserves thorough investigation, including consideration of EEG monitoring during sleep to detect a possible electrical status epilepticus during slow-wave sleep (ESES). **18. If I have prescribed fluoxetine to a teenager with depression but no history of epilepsy and he or she has a seizure, what should I do?** Indeed, at least at the group level, SSRIs are protective against seizures rather than precipitating seizures. Therefore, the recommendation will be to continue with the SSRI, unless there is a clear indication for not doing so. **OCD** **19. What steps do you take in medicating OCD in children and young people?** If there is little shift in the core OCD symptoms after three months at high dosage of an SSRI, cross titrating to a second SSRI is recommended. If this second trial, once again at high dosages, is ineffective after an additional 3 months, clomipramine or more commonly augmentation with low dose antipsychotic medication should be considered. **20. What is the approach to the use of atypical antipsychotics in OCD?** Only approximately one-third of treatment-resistant cases of OCD show an additional response to adding low dose antipsychotics to the SSRI. It is unlikely that an increase in dose of antipsychotic will bring additional benefit if low doses of antipsychotic have not been effective in terms of augmentation. **PERSONALITY DISORDERS** **21. What drug treatment should I use if a patient has BPD?** There is no evidence that any medication is effective for the core symptoms of BPD in adults or adolescents, and limited evidence that medication is effective for co-morbid mental illness in adults with BPD, with no evidence for this in adolescents. Instead, the mainstay of treatment of BPD (in all age groups) is based on psychological therapy programmes, in particular dialectical behaviour therapy and mentalisation-based treatment **PSYCHOTIC SPECTRUM DISORDERS** **22. Can I diagnose and treat schizophrenia in adolescents who are consuming illicit drugs?** If diagnostic criteria for schizophrenia are met in an adolescent with a substance use disorder, both diagnoses should be made. There is no evidence-based guidance on choice of antipsychotic on adolescents with schizophrenia and a co-occurring substance-use disorder. Long-acting injectable antipsychotics have shown some efficacy in adults with these conditions and may be an option in older adolescents. Antipsychotic treatment may also be considered if symptoms of psychosis persist in adolescents who are not able to maintain abstinence. **23. How do I treat an adolescent with treatment-resistant schizophrenia?** After ruling out possible causes of lack of effectiveness of prescribed treatments (including wrong diagnosis, poor adherence and suboptimal dose), clozapine should be considered after failure to respond adequately to a trial of two different antipsychotics. **24. How do I manage weight increase related to the use of antipsychotic medications in children and adolescents?** Treatment options include switching to a less orexigenic/metabolically adverse antipsychotic (such as potentially lurasidone and brexpiprazole, even though data are still limited), adjunctive behavioural treatments, and adjunctive pharmacologic interventions, including metformin, which however in general leads to quite modest weight reduction. Education about strategies to enhance appropriate nutritional style and physical activity, and regular weight monitoring, are key. **TICS/TOURETTE’S SYNDROME** **25. Is it best to replace methylphenidate with guanfacine or use it as adjunct when ADHD is present but tics require treatment as well?** If methylphenidate is already effective in treating ADHD symptoms, but tics worsen and/or require treatment, guanfacine may be added. In cases of mild-to-moderate ADHD symptoms with co-existing tics, and/or if methylphenidate is only partially effective or poorly tolerated, discontinuation of methylphenidate and switching to guanfacine may be considered. If tics improve with guanfacine alone but ADHD worsens, then methylphenidate may be restarted. In case of moderate–to-severe ADHD with tics, ADHD symptoms are likely to require treatment with psychostimulants which are the first-line treatment choice for ADHD. **26. When would I consider antipsychotics more appropriate than clonidine/guanfacine when treating tics in children and young people?** Because of the more serious adverse effects of antipsychotics but similar efficacy for tics compared to noradrenergic drugs, it is recommended that noradrenergic drugs (i.e., clonidine and guanfacine) are used first-line. However, antipsychotic medications may be more beneficial in certain individuals and may be more effective on some of the behavioural symptoms associated with tic disorders and on the common comorbidities such as ASD and OCD. **27. When clonidine is being used in higher doses is it best to go beyond treatment window or consider a switch to guanfacine? If switching, is it best to reduce and withdraw Clonidine before initiating Guanfacine or can I cross taper?** In individuals who tolerate doses higher than 300 mcg/day of clonidine there should be regular monitoring of blood pressure and pulse. In the case of comorbidities where behavioural modification is desired, guanfacine seems to have a better effect. When switching from clonidine to guanfacine, it is recommended to decrease clonidine by 25 mg every 3–5 days, stop clonidine and then start guanfacine. However, if this is not practicable given the severity of tic/sleep disturbance/behavioural symptoms, cross-taping is recommended by reducing clonidine to 100 mcg daily before initiating guanfacine at 1 mg daily and then reducing clonidine by 25 mcg every 3 days before guanfacine is increased as necessary.

ADHD: attention deficit hyperactivity disorder; ASD: autism spectrum disorder; BD: bipolar disorder; BED: binge eating disorder; BPD: borderline personality disorder; CBT: cognitive behavioural therapy; ESES: epilepticus of slow-wave sleep; FDA: Food and Drug Administration; GP: general practitioner; OCD: obsessive-compulsive disorder; SSRI: selective serotonin reuptake inhibitor.

## Attention-deficit/hyperactivity disorder

### Can I use doses of psychostimulants beyond the maximum licensed or recommended ones?

The maximum recommended doses of psychostimulants (which act by inhibiting the reuptake of dopamine and norepinephrine and, for amphetamines, inducing their release) for the treatment of attention-deficit/ hyperactivity disorder (ADHD) in guidelines or formularies may be higher than the maximum licensed doses by regulatory agencies such the Food and Drug Administration (FDA) or the European Agency. More specifically, the maximum licensed dose of methylphenidate for children (except for osmotic release and prolonged release formulations, see below) is 60 mg/day, while the British National formulary (BNF) (Joint Formulary Committee, 2022) recommends a dose of up to 90 mg/day, under the direction of a specialist. For osmotic-release (Concerta® XL, Janseen Cilag, High Wycombe, Buckinghamshire, UK) and prolonged-release (e.g. Xaggitin® XL, Ethypharm, High Wycombe, Buckinghamshire, UK and Delmosart® prolonged-release tablet, Accord UK Ltd, Barnstaple, Devon, UK) formulations of methylphenidate, the maximum license dose is 54 mg/day, but the BNF mentions a maximum of 108 mg/day for Concerta® XL, in line with other clinical guidelines, for example, those from the Canadian ADHD Resource Alliance (caddra.ca). For lisdexamfetamine, both the maximum licensed *and* the BNF recommended dose is 70 mg/day.

Many prescribers will use doses above the maximum licensed ones. Some of them will also use doses beyond the maximum recommended in guidelines/formularies. Currently, there is no solid, meta-analytic evidence to inform if, and to what extent, doses beyond the recommended ones are safe and bring additional efficacy/effectiveness. Some experts agree that, while it should not be a standard, routine practice, using doses beyond the maximum recommended ones could be considered when the patient has presented with a partial response, there is only some degree of improvement at the maximum recommended dose, tolerability is good, and the aim is to optimise the response (Cortese et al., 2021). This could be considered particularly in patients with overweight when a partial response was obtained at the licensed dose. If doses beyond those recommended are used, a careful monitoring of blood pressure, heart rate, height and weight should be implemented.

### What shall I do if a patient does not respond to psychostimulants?

Following poor response to two psychostimulants (methylphenidate, MPH and amphetamines, AMPH – including lisdexamfetamine, LDX), some clinicians would consider second- or third-line approved compounds, unlicensed medications for ADHD, or combinations of different agents. However, a number of factors should be assessed before switching to alternative medications or using polypharmacy. Indeed, it should be highlighted that the majority of patients with ADHD respond to one or both classes of psychostimulants, when used properly. A comparative review (Arnold, 2000) of randomised controlled trials (RCTs) found that approximately 41% of children treated with immediate-release stimulants responded equally well to AMPH or MPH, 28% responded better to AMPH, 16% had a better response to MPH and 15% did not respond to either medication. A more recent review concluded that approximately 91% of individuals with ADHD respond to either or both class of stimulants (Hodgkins et al., 2012). (We note that, as RCTs often exclude participants with specific comorbidities that decrease the rate of response, the response rate in patients seen in daily clinical practice may be lower.) Therefore, before considering alternative agents and/or polypharmacy, the following should be considered: (a) Have I titrated properly? While some patients will respond well to low or moderate doses, others will need higher ones, regardless of their age and weight. Of note, meta-analytic evidence from flexible-dose trials for both MPH and AMPH demonstrated increased efficacy and reduced likelihood of discontinuations for any reason with increasing stimulant doses (Farhat et al., 2022); (b) Is this drug/preparation working well at any times during the day or do I need to change the dose or preparation to get a more comprehensive coverage? Sometimes, poor response may be detected only in specific periods of the day, when the medication effect has worn off; (c) Am I targeting the right symptoms? Psychostimulants are in general highly efficacious/effective on the core symptoms of ADHD (i.e. inattention, hyperactivity, impulsivity) (Cortese, 2020), not necessarily on other problems (e.g. oppositional behaviour/emotional dysregulation); (d) Is the patient showing tolerance? Evidence from clinical studies, for example, Vitiello et al. (2001) shows the need to increase the dose of stimulants over time in order to maintain therapeutic response. Additionally, neuroimaging studies (e.g. PET studies; Wang et al., 2013) point to an increase in dopamine reuptake receptors in adults with ADHD treated for up to 12 months with stimulants. This evidence suggests that tolerance may happen during treatment with psychostimulants, even though more research is needed to gain a better understanding of the exact percentage of patients who develop tolerance, their clinical characteristics and how to manage tolerance effectively. Some experts, for example, Taylor (2019) suggest decreasing the dose or temporarily (for a few weeks) stop the psychostimulant to overcome the tolerance issues; (e) What else is going on in patient’s life/family life? A comprehensive formulation, beyond diagnosis, is key here; (f) Have I missed any comorbidity? Some comorbidities, for example, autism spectrum disorder (Rodrigues et al., 2021), are associated with lower chances of response; (g) Is the diagnosis correct?

Only after all these aspects have been assessed, the prescriber should consider: (1) second-line medications (atomoxetine – which selectively inhibits the norepinephrine transporter, and guanfacine – that selectively stimulates alpha-2 adrenergic receptors, or clonidine, that selectively stimulates alpha-2A adrenergic receptors); (2) augmenting agents (guanfacine or clonidine XR); (3) other agents, under specialistic advice/supervision, for which RCTs provide preliminary evidence of efficacy (e.g. bupropion, a non-competitive antagonist of nicotinic acetylcholine receptors) (Cortese et al., 2021).

### Does the concomitant use of cannabis impact on the effectiveness and tolerability of psychostimulants?

The evidence on the effectiveness of psychostimulants in marijuana users and the tolerability of the combination psychostimulants–marijuana is limited. In a RCT of OROS-methylphenidate and cognitive behavioural therapy (CBT) in youths with substance use disorder (in the majority of the cases (66%), marijuana), there were no significant differences, in terms of efficacy, between participants assigned to OROS-methylphenidate plus CBT and those in the placebo plus CBT, when considering the primary outcome (scores on the ADHD-RS completed by the clinicians), even though ADHD-RS scores rated by parents were significantly lower in adolescents treated with OROS-MPH + CBT compared to those in the placebo + CBT arm at 8 and 16 weeks. The tolerability of OROS-methylphenidate was not substantially different compared to what it would be expected with OROS-methylphenidate alone (Riggs et al., 2011). Another RCT showed addictive effects of MPH and tetrahydrocannabinol on heart rate, but not on systolic and diastolic blood pressure (Kollins et al., 2015). Therefore, even though there is no solid evidence, psychostimulants may be overall well tolerated, but their effectiveness may be decreased by the concomitant use of marijuana. This should be discussed with the patient at the initial assessment and during the follow-up visits. If a psychostimulant is prescribed, it is crucial to monitor its possible misuse carefully. The chances of misuse can be decreased by using long-acting as opposed to immediate-release formulations.

It should be noted that a subgroup of youths who use marijuana also tend to misuse other illicit substances, which may further impact on the tolerability of psychostimulants. Therefore, prescribers should comprehensively screen for the use of a variety of illicit drugs.

## Anxiety and depressive disorders

### What do I do if a young person has depression and has not responded to two SSRIs and CBT?

National Institute for health and Care Excellence (NICE) guidelines currently recommend CBT as a first-line treatment for moderate-to-severe depression, fluoxetine, a selective serotonin reuptake inhibitor (SSRI), as a first-line antidepressant and, if that is not effective, sertraline or citalopram (NICE, 2019). There is limited evidence for other treatments beyond this, other than interpersonal psychotherapy (Zhou et al., 2015), which is often not available.

The first thing to do when a patient is treatment-resistant is to revisit the formulation and diagnosis. Is this really depression, or does the pattern of symptoms since you started seeing the patient suggest a personality disorder as a more appropriate diagnosis? Are there any social factors that have not been addressed?

Then review whether the patient has indeed had all the treatments to which they appear to be ‘resistant’. Did they stop fluoxetine after a few days of mild side effects and would they be prepared to try again? Did they have a good number of sessions of evidence-based psychological therapy from a fully trained and supervised therapist? If they stopped after a few sessions, was this due to a problem in the therapy or the therapist and would they consider trying a different therapist?

If the patient truly does have depression, and you have tried all the evidence-based treatments, there is no evidence to guide you. It would be reasonable to try two SSRIs up to the maximum tolerated dose, within BNF limits (Joint Formulary Committee, 2022): although there is no evidence in favour of or against this, there is some evidence for a dose increase in adult depression; and there is plenty of safety data from paediatric OCD studies which use high doses of SSRIs. If the patient is nearly 18, it may be reasonable to try antidepressants shown to work in studies of 18–64 years old but not in studies of 12–17 years old, such as mirtazapine (adrenergic alpha-2 auto receptors and heteroceptors antagonist and 5-HT2 and 5-HT3 receptors blocker) or venlafaxine (serotonin and norepinephrine inhibitor); caution should be used with the latter due to possible increased risk of suicidal ideation, and for that reason it is especially important to seek peer advice. It may be worth trying an augmenting agent that has evidence in adults such as lamotrigine, an atypical antipsychotic, lithium, or l-thyroxine (Bschor and Bauer, 2006; Cantù et al., 2021; Goh et al., 2019; Kalra and Balhara, 2014).

In all cases, it is important to discuss and document potential benefits and harms with patients and their families, and the lack of robust evidence or marketing authorisation for any drug treatment other than fluoxetine 20 mg once daily. The practicality of blood monitoring in local services may imply that it is not feasible to prescribe antipsychotics and (especially) lithium. It is also important to discuss marketing authorisation regulations for potential antidepressants in your own country (for example in the United Kingdom, marketing authorisation for adolescent depression is only present for fluoxetine). If you are unsure, it is important to discuss cases with your consultant peers and to document this.

### If a patient has severe side effects (such as suicidality) on one SSRI, is it safe to try a second SSRI?

While the majority of adolescents given an SSRI have mild, transient side effects, a small number of patients have rarer and more dangerous side effects, including suicidal thoughts (Bridge et al., 2007), homicidal thoughts, and clotting abnormalities (Lake et al., 2000). Many patients and their families (and their prescribers) will choose to stop the medication in this situation.

There is then the question of whether another SSRI should be used. There is no published evidence regarding this. However, in consensus discussions, several psychiatrists have tried the patient on a second SSRI and this has not led to the same side effect. It is likely the patient will have increased risk of, for example, suicidality on a second SSRI compared to a patient who has never had an SSRI, but it could be worth trying, especially in more severe depression which itself carries significant suicide risk. As always, it is important to discuss potential benefits and risks with patients and families and make decisions jointly. It is essential to document these discussions. It is also especially important to follow these patients up regularly when starting a second SSRI and have a clear plan in place for what is to be done if these side effects recur. If unsure, discuss the case with consultant peers and document this.

It is also important to take into account the pharmacological profile of the first and second SSRI. Among the SSRIs, sertraline and citalopram/escitalopram have significantly higher 5-HT specificity than fluoxetine. So, if sertraline caused dangerous side effects, it would be better to try the more different fluoxetine than citalopram as second line.

### I see an adolescent with an anxiety disorder who a GP has started on propranolol. What should I do?

SSRIs are by far the most effective medication for anxiety disorders in children, adolescents and adults (Ipser et al., 2009). General practitioners (GPs) are often (and rightly) cautious about prescribing SSRIs in children and adolescents. This leads some GPs to prescribe propranolol (a beta blocker) instead, for anxiety disorder. There are two problems with this: 1) propranolol is not an effective treatment for anxiety disorders (Steenen et al., 2016); 2) propranolol has significant side effects and is particularly dangerous in overdose. Its use seems to have stemmed from the fact that it can reduce the physiological symptoms of anxiety. However, in most cases, doing this does not reduce the feelings of anxiety. Therefore, it is much better to use SSRIs, which are both effective and safer. It may be better still to offer CBT, which has similar efficacy to SSRIs (Walkup et al., 2008), but is more likely to keep the patient well long-term, and has no physical side effects.

If we see a patient with an anxiety disorder who is taking propranolol, we should sensitively explain that it is not effective, and offer them an SSRI instead if they would like medication. If the child/adolescent has recovered from the anxiety by the time we see them, it is still worth stopping the propranolol as they may no longer need it. It is also important to educate the GP that propranolol is neither safe nor effective for anxiety disorders.

One possible exception is children/adolescents with autism spectrum disorder (ASD) and anxiety. Some ASD experts report that children with ASD are much more sensitive to internal somatic stimuli, and if they have pronounced physical symptoms of anxiety, reducing these with propranolol may make a big difference and reduce overall anxiety. However, there is no clear evidence for this, and it is probably more appropriate to try an SSRI first.

## Autism spectrum disorder (ASD)

### Can I use SSRI medication to manage repetitive behaviours in a child with a diagnosis of ASD?

Repetitive behaviours are a core diagnostic component of ASD and, consequently, commonly observed among individuals with autism of all ages. These behaviours can take different forms: broadly speaking, while for some they comprise ‘functional’ behaviours, such as the pursuit of interests, for others they may be less purposeful, such as ritualistic and repetitive motor behaviours. The decision to treat is fundamentally determined by the degree to which the behaviour impacts on wellbeing and/or the ability to develop other skills.

Treatment of repetitive behaviours will largely be from a psychological/behavioural perspective. In some circumstances, however, these may be ineffective, or otherwise difficult to implement; in such cases, medication may be considered. The Cochrane Review of 2013, which considered the use of SSRIs in the management of ‘core’ symptoms of ASD, found no evidence to support their use for ritualistic behaviours (Williams et al., 2013). A more recent systematic review similarly concluded that there is no evidence for the use of SSRI or other medications in the management of ritualistic behaviours (Yu et al., 2020).

This notwithstanding, when examining individual studies, there is certainly some evidence to suggest that certain SSRIs (Carrasco et al., 2012), guanfacine (Politte et al., 2018), risperidone- a D2, 5-HT2 and NE alpha-2 receptor antagonist (D’Alò et al., 2021; McDougle et al., 2005) and buspirone – a 5-HT1A receptor partial agonist (Chugani et al., 2016) may be effective. Given these data, it may be reasonable to consider an SSRI when other non-pharmacological treatment options have failed given that these are used widely in clinical practice and so safety data are available, although some ASD clinical trials have raised the higher risk of side effects in this population (see Williams et al., 2013; Yu et al., 2020 and references to individual studies therein). If SSRI are ineffective, or poorly tolerated, risperidone, guanfacine or buspirone may be considered as an alternative; however, particular caution is advised in relation to their use, which must be under specialist supervision.

At the outset it is essential that any trial of medication has (i) a clear titration schedule and (ii) well-defined outcome measures. It is important to bear in mind that medication may take several months to alleviate symptoms. In accordance with the Stopping The Over-Medication of children and young People (STOMP with a learning disability, autism or both) and Supporting Treatment and Appropriate Medication in Paediatrics (STAMP) frameworks (https://www.england.nhs.uk/wp-content/uploads/2019/02/STOMP-STAMP-principles.pdf), medication should be discontinued if it does not help, and, among responders, should be monitored and discontinued when appropriate.

### What are my options in the management of aggression in a child with ASD?

Aggression is one of the most frequent reasons for referral to specialist services for a child with ASD, particularly among those children with ASD who have co-morbid intellectual disability. From the outset, a multidisciplinary approach is crucial in any such assessment to identify the relevant underlying factors so these can be managed (Howes et al., 2018; Volkmar et al., 2014). Aggression is not a diagnosis; instead, it is a mechanism for the communication of need, discomfort or distress. The clinical team must identify the relevant biological, psychological and social predisposing, precipitating and perpetuating factors so that these can be managed accordingly. In rare instances, major life events, or factors such as constipation or pain, are key, but more commonly multiple factors are relevant. Aggression may also be a symptom of an underlying psychiatric diagnosis such as depression or psychosis, which should be managed accordingly. Despite the recommendations made below, it is important to bear in mind that psychotropic medication can itself result in agitation, anxiety and mood disturbance, and so careful attention needs to be paid to the possibility that symptoms may be getting worse *as a result of* medication.

In the management of aggression, medication may be deemed an appropriate option in a number of different situations. For example, in the acute situation where it would be difficult or potentially unsafe to introduce behavioural therapy, medication can offer the possibility of reducing the severity of symptoms and thereby improving engagement in psychological interventions. Either risperidone (1–2 mg) or aripiprazole – a D2 and 5-HT1A receptor partial agonist (5–10 mg) can be used for this purpose, both having shown efficacy and safety among autistic individuals (Hirsch and Pringsheim, 2016; McCracken et al., 2002; McQuire et al., 2015) at lower doses compared to when they are used as antipsychotics (of note, at these lower doses, serotonergic antagonism, rather than dopamine antagonism, predominates). Both have FDA approval for agitation in ASD. If used for this purpose, it is important that it is discontinued once behavioural work is underway. Less is known about the acute (and maintenance) use of other (so-called) antipsychotics (D’Alò et al., 2021; McQuire et al., 2015). Similarly, benzodiazepines – positive allosteric modulators of the GABA-A receptor-are sometimes used but there are no data.

In other situations, the consideration of medication may be raised when behavioural therapy has been unsuccessful or only partially successful. It is always important to consider the reasons for this lack of success, including revisiting the possibility of underlying medical causes (pain, constipation, metabolic, allergic and so forth). As in the acute situation, risperidone or aripiprazole may be introduced, with the expectation that the child is likely to remain on this over a period of 6 months or more. It is important that clinical monitoring takes place according to local policy, which in the United Kingdom may vary between NHS Trusts (Howes et al., 2018). The FDA recommends monitoring according to known risk factors (e.g. cardiovascular risk factors) and emerging unwanted symptoms such as polydipsia and polyuria. The NICE Clinical Knowledge summary ‘What monitoring is required?’ (https://cks.nice.org.uk/topics/psychosis-schizophrenia/prescribing-information/monitoring/) in relation to risperidone use in children advocates monitoring for hyperprolactinaemia 6 months after starting treatment, and then every 12 months. Collection of blood may not always be possible, in which case the decision to continue medication must be made in discussion with the patient and family and clearly documented. Other (so-called) antipsychotics may also be used second line, but less is known about their use in this population. Similarly, benzodiazepines can have both a calming and anxiolytic effect, but there are no data on their use in this population. There is no evidence that SSRIs are effective for aggression, and no evidence for the use of mood stabilising treatments (unless of course a specific relevant diagnosis of bipolar disorder (BD) has been made). There is also no evidence for using different medications in combination.

Deciding on when to discontinue medication is also important, and a decision must be taken early in treatment. It may be reasonable to begin discontinuation at the 6-month point, or sooner for some. However, in reality other factors will influence this decision, such as whether or not the child has structure during the day (less likely during school holidays) and whether there is additional support available.

### What are my pharmacological choices for the management of aggression in a child with ASD when standard options seem to have failed?

For some children, behavioural interventions combined with ‘first line’ medications such as those suggested above fail to alleviate symptoms. There may be several reasons for this, and it is crucial at the outset that these are explored. The diagnostic formulation may have missed an important aetiological factor, or previously identified triggers or perpetuating factors may have been inadequately managed. For example, an environment with certain characteristics, such as routine, predictability and appropriate level of stimulation, may not be made available. Or there may be an absence of adequately trained support staff and staff may fail to follow behavioural recommendations. In these circumstances it is crucial that medication does not become the perceived ‘solution’. It is unethical for the psychiatrist to be expected to medicate where services are unable to meet needs.

On the other hand, there will be situations where challenging behaviour continues despite attention to all identified aetiological factors. Such situations are most likely to arise in the context of non-verbal autistic children who are severely or profoundly cognitively impaired. While it remains important to continue to work towards a great understanding of the behaviour’s underpinning, this is often very challenging and medication may thus be considered as a least restrictive, best interest option in consultation with decision makers. Additionally, the psychiatrist should discuss treatment-resistant cases with their peer group, and consider seeking a second option.

Other medications than those already discussed above have much less evidence for efficacy. Other (so-called) antipsychotics such as quetiapine – receptor antagonist (D2 and 5-HT2) or olanzapine – D2 and 5-HT2 receptor antagonist – are certainly reasonable options, although they have not been studied robustly in this population (Chugani et al., 2016; D’Alò et al., 2021; Hirsch and Pringsheim, 2016; Howes et al., 2018; McCracken et al., 2002; McQuire et al., 2015; Volkmar et al., 2014). Consequently, these medications must be used with caution, including low initial dose, slow titration and regular monitoring. Alternatively, benzodiazepines such as lorazepam, diazepam or clonazepam may be considered, but the same caution is advised. The combination of an antipsychotic with a benzodiazepine may also be considered, but the aim must always be to achieve symptomatic relief without sedation, such that the child can continue to engage in activities. If a benzodiazepine is prescribed, it must always be only used in the short term.

SSRIs do not have a role to play in the management of aggression, and their use should be limited to the management of mood disorders, anxiety and, in some circumstances, repetitive and ritualistic behaviours as discussed above. Clozapine-D2, 5-HT2 and NE alpha-2 receptor antagonist are sometimes used to manage aggressive behaviour in autistic people, but this is based on limited evidence in adults (Rothärmel et al., 2018). There are no data available for its use for this purpose in the paediatric population. There is no evidence for other agents, such as mood stabilisers or GABAergic agents (pregabalin).

## Bipolar disorder

### How does one distinguish ADHD from Bipolar Disorder (BD)?

As a neurodevelopmental disorder, the features of ADHD are usually present early in childhood and often cause functional impairment by the time a child is in primary school, whereas BD usually presents in teenage years with peak onset described as between 15 and 19 years (NICE, 2014). Another factor that helps distinguish ADHD from BD is the course of symptoms as in ADHD the symptoms are pervasive and generally chronic over time (with some variation in severity); BD presents with an episodic course of symptoms (in children and adolescents these episodes may have shorter durations than those seen in adults) with periods of euthymia interspersed. Lastly, ADHD is a disorder of attention, motor activity and impulsivity whereas BD is a disorder of mood. Of course, these disorders may occur co-morbidly as well, and if this is the case appropriate management strategies should be employed.

### How does one distinguish Borderline Personality Disorder (BPD) from BD?

BD is a disorder of mood characterised by discrete mood episodes. Periods between the mood episodes also described as euthymia are usually characterised by stable psychosocial functioning. Borderline personality disorder (BPD), also known as emotionally unstable personality disorder (EUPD), is a personality disorder characterised by a long-term pattern of unstable interpersonal relationships, distorted sense of self and marked affective instability. The affective instability is often triggered by environmental factors and usually lasts hours to days, unlike the mood episodes in BD which last weeks to months. Other clinical variables that help distinguish BD from BPD include: response to pharmacology in BD, a family history of BD, and the fact that BPD is often associated with significant early life trauma. When in developing individualised treatment approach, adopting a dimensional approach in addition to a categorical approach, is beneficial particularly when these conditions are co-morbid.

### How does one distinguish treatment-emergent affective switch (TEAS) from behavioural activation when using antidepressants and what is the management?

Both phenomena can occur quite soon after the initiation of antidepressants. The International Society for Bipolar Disorders Task Force recommends using the term ‘treatment-emergent affective switch’ (TEAS) instead of antidepressant-induced switch to emphasise association of (hypo-)manic symptoms when treating with antidepressants without implying causality. Different researchers operationalise the time frame in which TEAS can emerge with Chen et al. (2022) using 8 weeks while others such as Truman et al. (2007) define this as 12 weeks. However, the task force also states that if symptoms occur within 2 weeks of initiating antidepressants, then to term it as antidepressant-associated TEAS.

In the activation syndrome, symptoms can include impulsivity, insomnia, restlessness, hyperactivity and irritability, while on the other hand antidepressant-induced manic switch presents with symptoms of a (hypo-)manic episode. Mild hypomanic symptoms are much more common than manic switching (Emslie et al., 2006) and it may not always be easy to differentiate a hypomanic switch from behavioural activation. Indeed, some scholars suggest that behavioural activation might represent subsyndromal manic symptoms or unrecognised BD, especially in young persons who have not been diagnosed (Amitai, 2015). However, other researchers highlight that manic spectrum symptoms involve a change in mood and behaviour with coexisting symptoms of grandiosity and euphoria which are not found in the SSRI-induced activation syndrome (Walkup, 2001). The treatment for a manic switch includes immediate discontinuation of the antidepressant (and possible use of antimanic agents) whereas a reduction of or discontinuation of antidepressants benefits the activation syndrome.

## Eating disorders

### What additional considerations are needed when prescribing in young people with eating disorders?

There are considerations that relate to the physical complications of eating disorders. These include cardiac risks secondary to malnutrition and electrolyte abnormalities secondary to malnutrition or purging. Low weight can cause bradycardia and prolong QTc interval, thus increasing the risk of arrythmia (Giovinazzo et al., 2019), as can some psychotropic medications, in particular haloperidol, a D2 receptor antagonist. Studies suggest olanzapine has little effect on QTc interval (Jensen et al., 2015) but the UK manufacturer advises caution on the basis that other antipsychotics have QT prolonging effects. Purging causes potassium depletion, and low potassium can also arise secondary to severe weight loss. Hypokalaemia can further prolong QTc interval as well as causing arrythmia in its own right. It is therefore good practice to perform an ECG before prescribing.

Another consideration is patient engagement and adherence with prescribing. Anxiety about, and sensitivity to, side effects need to be addressed directly before initiating medication, in particular fear of weight gain secondary to antipsychotics. Patients should be reassured that the small doses of antipsychotics such as olanzapine typically used will have a minimal impact on appetite, that use of antipsychotics is short term (months rather than years) and that weight can be managed by adherence to meal plans during therapy. The primary purpose of prescribing antipsychotic medication in patients with anorexia nervosa is to reduce overwhelming arousal during meal times and not directly to cause weight gain.

### Should comorbidities in young people with eating disorders be treated separately?

Comorbidities in eating disorders are common – up to 55% of children and adolescents presenting with an eating disorder have a comorbid disorder at presentation (Simic et al., 2022; Swanson et al., 2011). The commonest are depression, anxiety (especially social anxiety), obsessive-compulsive disorder (OCD) and neurodevelopmental disorders such as ASD and ADHD. Three important considerations are warranted when considering whether to treat the comorbidity. First, eating disorders can mimic or accentuate comorbidities, which are corrected as symptoms resolve. An example is the quasi-autistic effects that occurs in starvation syndrome, such as difficulties in set shifting and enhanced central coherence (Pender et al., 2014). Second, effective psychological treatments for eating disorders can result in improvements in other areas. For example, family-based treatment for anorexia nervosa, once malnutrition has been corrected, involves supporting dialogue around adolescent challenges and anxieties, such as difficulties in school or in interpersonal relationships, in a supportive family context. Clinical trials show resolution in anxiety and depression as well as the eating disorder at the end of treatment for the majority of subjects (Agras et al., 2014). Finally, studies suggest that some pharmacological interventions are ineffective during the acute phase. For example, antidepressants show limited efficacy in the context of malnutrition and it has been suggested that this decrease/loss of antidepressant efficacy is due to starvation-related structural and biochemical/pharmacologic changes in the brain (Marvanova and Gramith, 2018).

However, in some patients, the history clearly suggests that untreated or undiagnosed mental disorder may have preceded onset of the eating disorder and even that the eating disorder functioned as a ‘solution’ to these feelings of anxiety or other negative affect. For example, OCD may remerge as anorexia nervosa recedes. If a patient is not responding to competently delivered treatment as expected, re-evaluation of the diagnosis and formulation and consideration of additional treatment strategies for comorbidity should be considered.

### How might treatment approaches differ in young people with an eating disorder in the context of neurodevelopmental disorder?

Neurodevelopmental disorders are common across mental disorders, and are especially associated with eating disorders. Studies report between 4 and 52.5% of participants with anorexia nervosa meet suggested clinical cut-off for ASD, with higher proportions in adult that in younger populations (Westwood and Tchanturia, 2017). This wide range reflects the heterogeneity in the diagnostic assessment across studies. ASD is also commonly comorbid with avoidant restrictive food intake disorder. Treatment adaptations for comorbid ASD are in development (e.g. the PEACE pathway; Tchanturia et al., 2020). The possibility of comorbid ASD is an important consideration when prescribing in terms of anticipated response, treatment duration and side effect sensitivity. In general, people with ASD are more likely to remain on medication and to be prescribed polypharmacy. What research there is, suggests autistic people may be more likely to experience side effects such as drowsiness, irritability and reduced activity (Persico et al., 2021).

ADHD is commonly comorbid in the context of bulimic and binge eating disorder (BED) (Nazar et al., 2016). Identification, assessment and treatment of comorbid ADHD may be helpful in addressing difficulties in impulsivity and self-regulation common to both disorders. The potential appetite suppressing effects of ADHD treatment should also be a consideration in evaluating the treatment options. Trials of lisdexamfetamine show favourable results compared with placebo on remission, change in body mass index (BMI) and binge eating in adults with BED (NICE, 2017). However, in trials, more people withdrew due to adverse events in the active arm, and there was a trend towards higher depression scores in the lisdexamphetamine arm compared with placebo. Lisdexamfetamine is not licenced in the United Kingdom for treatment of BED, but is licensed for treatment of ADHD. No pharmacological trials in young people with BED have been conducted as yet.

## EPILEPSY (in differential diagnosis or comorbid with mental conditions)

### What is the role of cannabidiol in the child with epilepsy and behavioural problems?

There are now sufficient high-quality studies to confirm the efficacy of cannabidiol in treating seizures in both adults and children (Devinsky et al., 2016; Lattanzi et al., 2018; Szaflarski et al., 2018). The mode of action of cannabidiol as an antiseizure medication remains uncertain. The efficacy and adverse effects are not very different from those of other relatively new antiseizure medications. Overall, it is not notably more effective or less effective. With regard to either beneficial or adverse behavioural effects, there is currently conflicting evidence, although with some suggestion of benefit (Arzimanoglou et al., 2020). A confounding factor is that seizure frequency can affect behaviour, implying that if the cannabidiol affects seizure frequency, it may be difficult to determine whether any change in behaviour is the result of a change in seizures or whether it is a direct result of the cannabidiol itself. There are also some important drug interactions; for example, cannabidiol raises clobazam blood levels. Further evidence will be required before definitive information on the effects of cannabidiol on behaviour in young people with epilepsy can be provided.

### If a child is having nocturnal episodes that are disturbing sleep, when should we be assessing for epilepsy as a possible cause?

In the absence of a history of daytime seizures, the likelihood of undiagnosed nocturnal seizures is less. However, there are certain types of epilepsy that present with seizures that occur only or primarily at night.

In particular, nocturnal seizures of frontal lobe origin can present in a way that can be inconsistent with what most people would consider as being typical of epileptic seizures (Nobili et al., 2014; Scheffer et al., 1995). Nocturnal frontal lobe seizures typically present with brief episodes that can occur multiple times in a single night, often with non-rhythmical movements. They are frequently accompanied by vocalisation and, in contrast to the situation for most other focal-onset seizures, it is often possible for the individual to recall the seizures afterwards. The recollection can be disturbing, sometimes being associated with a subjective difficulty in breathing, which could be misdiagnosed as asthma. A careful history will usually distinguish nocturnal frontal lobe seizures from other nocturnal episodes. For example, the characteristics of nocturnal frontal lobe seizures can be distinguished clearly from night terrors, through careful history taking. A video of the nocturnal seizures, perhaps using an infrared camera, can assist in the diagnostic process. Nocturnal frontal lobe seizures classically respond to treatment with carbamazepine; they can also respond to other antiseizure medications, such as levetiracetam.

Children with nocturnal tonic (stiffening) seizures generally have a background history of epilepsy. Because they are brief, nocturnal tonic seizures may be difficult to distinguish from normal nocturnal movements but video EEG monitoring can be diagnostic. Brief tonic seizures can occur many times in a single night, sometimes more than 100 times. Such frequent seizures almost certainly affect daytime alertness and performance.

The other nocturnal epilepsy-related phenomenon that is of major importance, although rare, is electrical status epilepticus of slow-wave sleep (ESES), otherwise known as continuous spike-wave of slow-wave sleep (Tassinari et al., 2000). The definition of ESES requires that more than 85%of the slow-wave sleep be replaced by spike-wave epileptiform discharges. Although most cases present against the background of established epilepsy, some cases present with no history of overt seizures. ESES can be accompanied by profound loss of skills, especially profound loss of verbal auditory comprehension, which can lead to complete loss of speech, in some cases. ESES accompanied by loss of speech is known as the Landau–Kleffner syndrome (Landau and Kleffner, 1957) or acquired epileptic aphasia. Several medication treatments have been advocated, with some degree of success. If antiseizure medication is not effective, (Mikati and Shamseddine, 2005), then the surgical procedure of multiple subpial transection (Morrell et al., 1995) should be considered. Although reports of the efficacy of this procedure are variable, it can be highly successful in allowing the aphasic child to speak again. Any child who loses skills for no apparent reason deserves thorough investigation, including consideration of EEG monitoring during sleep. If this reveals ESES, then prompt treatment should follow. Children with acquired aphasia, understandably, may have major behavioural difficulties, including autistic features, emphasising the importance of effective, prompt and appropriate management (Besag et al., 2016).

### If I have prescribed fluoxetine to a teenager with depression but no history of epilepsy and he or she has a seizure, what should I do?

Both animal work and the landmark paper by Alper et al. (2007) analysing data on people with depression, strongly suggest that SSRIs are protective against seizures rather than precipitating seizures. A cursory examination of the data could result in misleading, incorrect conclusions. Depression itself is a risk factor for having seizures. People taking SSRIs for depression are more at risk of having seizures than the general population, which might lead to the incorrect conclusion that the SSRIs have increased the risk of having seizures. This is because the comparison is the wrong one. If a group of people with depression taking SSRIs is matched with a group of people *with depression* not taking SSRIs, then those taking SSRIs have a very much lower risk of having seizures, as confirmed by the Alper et al. review.

In brief, if someone taking SSRIs for depression has a seizure, the situation should be discussed fully with the young person/family. It is unlikely that the SSRI will have precipitated the seizure and it is even possible that the SSRI might have some protective benefit against further seizures. The family should be provided with accurate information on which to make a decision. Usually, the recommendation will be to continue with the SSRI, unless there is a clear indication for not doing so. If it is a single seizure with no associated clinical signs or symptoms that might cause concern, such as, for example, a new abnormality on neurological examination, it is usually not necessary to refer to paediatrician or neurologist. If further seizures occur, full assessment for epilepsy is recommended.

## Obsessive-compulsive disorder

### What steps do you take in medicating OCD in children and young people?

Much of the information held in NICE guidance for the initiation of treatment of OCD are still based on strong evidence (NICE, 2005). When choosing to initiate medication, as part of stepped care, it is important to have a clear narrative for your patient and their family about the typical need to increase to a high dose of SSRI. Meta-analytic evidence from of OCD treatment trials largely emanates from adult research but still emphasise the need to use a moderate-to-high dose of medication (Bloch et al., 2010). From clinical experience, most adolescents with OCD are able to tolerate weekly increments of medication, towards a high dose, for example, fluoxetine 60 mg daily. Understandably, young people with OCD are often very keen to see immediate effects of medication on their OCD symptoms. This is not realistic and a treatment trial for OCD should be around 3 months (NICE, 2005). It can be important to discuss the fact the SSRI may bring additional secondary benefits, such as improvements in mood and generalised anxiety, which are otherwise very common comorbidities. This can improve engagement and help longer term commitment to the OCD treatment. These gains for the young person’s mental state are also individually important.

Ideally measuring response to treatment with a standardised measure can be helpful to track change. If there is little shift in the core OCD symptoms after 3 months at high dosage of an SSRI, cross-titrating to a second SSRI is recommended. A full 3-month trial, again at high dosing is important. It would be after a second high dose trial that one would consider a trial of clomipramine or, more commonly, to consider augmentation with low dose antipsychotic medication.

### What is the approach to the use of atypical antipsychotics in OCD?

Patients who fail to show a good treatment response to SSRI medication may respond to the addition of a low dose antipsychotic medication to the SSRI therapy (Kayser, 2020). It is important to note that the evidence for this intervention is purely as an augmenting agent and that OCD will not respond to treatment with atypical antipsychotics alone. The research supporting this emanates from studies in adult-age patients and of the OCDs, it is only OCD and not body dysmorphic disorder that have been investigated.

In considering this intervention in children and young people, it is important that all the typical provisions and considerations be in place for prescribing and monitoring antipsychotics. It is also important to have a shared understanding with the young person and their family about the prospects for improvement. The data supports the use of low dose antipsychotic augmentation of an SSRI only. Approximately one-third of treatment-resistant cases of OCD will show an additional response to adding low dose antipsychotics to the SSRI (Bloch et al., 2006). It is important when starting this intervention to adopt an open and transparent approach. Two-thirds of patients will not show an improvement. It is therefore important to set a time limit for this intervention of around 2–3 months and then discontinue the intervention if there is no response. It is ideal that the intervention is monitored with an appropriate OCD measure, such as the Children’s Obsessive Compulsive Inventory (Shafran et al., 2003) or the Children’s Yale-Brown Obsessive Compulsive Scale (Scahill et al., 1997). Often clinicians can feel tempted to increase the dose of atypical antipsychotic, rather than being clear that if the patient is going to be in the minority that see this effect, it is likely to be with low dose atypical antipsychotic augmentation of the SSRI. It is often important to be clear to declare the trial of treatment as ineffective and to discontinue.

When treatment for OCD have been suboptimal, children, young people and their parents often ask for additional switches of medication. This can be an important moment in clinical practice, to discuss efforts around psychological therapy. Where medications have not brought about the required improvements, young people and their families need to understand that multiple further switches of psychopharmacology are unlikely to be helpful. It can be in these moments that one can renegotiate to redouble efforts around CBT for OCD.

## Personality disorders

### What drug treatment should I use if a patient has Borderline Personality Disorder (BPD)?

Personality disorders can and do occur in people under 18 and no diagnostic criteria say they cannot. Patients can find having an accurate diagnostic label helpful; the correct diagnosis helps clinicians to give appropriate treatments and the correct diagnosis leads to a more realistic prognosis being given (Kaess et al., 2014). Making such a diagnosis is not straightforward, given the complexity of adolescence, and is often better done over multiple assessments; it is also easier to do if the adolescent is older. The majority of evidence on personality disorders is for emotionally unstable/borderline PD.

There is no evidence that any medication is effective for the core symptoms of BPD in adults or adolescents. There is limited evidence that medication is effective for co-morbid mental illness in adults with BPD and no evidence for this in adolescents. Instead, the mainstay of treatment of BPD (in all age groups) is complex and integrates psychological therapy programmes. In particular dialectical behaviour therapy and mentalisation-based treatment have been found to be effective in adolescents with BPD although studies were limited by including mainly girls and only including the subset of patients with BPD who were happy to be in a treatment trial (Rossouw and Fonagy, 2012; Wilkinson, 2018). It is also essential to address social factors (e.g., current abuse, educational issues, stability of placement) in helping these patients.

## Psychotic spectrum disorders

### Can I diagnose and treat schizophrenia in adolescents who are consuming illicit drugs?

Schizophrenia has a rise in prevalence during adolescence, which is also the time when drug consumption rises. Cannabis, particularly high-potency cannabis, is a common precipitant or contributor to psychosis in young people (Di Forti et al., 2019). Youth experiencing psychosis also frequently misuse substances, which makes the differential diagnosis between substance-induced psychosis and primary psychotic disorders challenging.

NICE guidelines advise inquiry about the use of alcohol and illicit drugs when assessing a young person with psychosis and, if present, about particular substances and patterns of use (NICE, 2011). Performing a drug urine test may be also useful in acute psychosis. In drug-induced psychosis, psychotic symptoms present in the context of acute intoxication or withdrawal from substances, with a gradual recovery and remission within 1 month of sustained abstinence (American Psychiatric Association, 2013), which may not always be possible to achieve. Regarding differential clinical features with primary psychotic disorders, later age of onset, fewer negative symptoms, greater insight and less frequent family history of psychosis have been reported more frequently in drug-induced psychosis than in primary psychosis (Beckmann et al., 2020), although differential diagnoses with primary psychotic disorders cannot be made based solely on them.

If diagnostic criteria for schizophrenia are met in an adolescent with a substance-use disorder, both diagnoses should be made. Integrated care, in which treatment for both disorders is provided simultaneously by the same clinician in community-based mental health teams, should be provided (NICE, 2011). There is no evidence-based guidance on choice of antipsychotic in adolescents with schizophrenia and a co-occurring substance-use disorder. However, this comorbidity complicates management and prognosis, increasing relapse rates and worsening medication adherence (Beckmann et al., 2020). Long-acting injectable (so-called) antipsychotics have shown some efficacy in adults with these conditions and may be an option in older adolescents (Coles et al., 2021). Antipsychotic treatment may also be considered if symptoms of psychosis persist in adolescents who are not able to maintain abstinence (Beckmann et al., 2020).

### How do I treat an adolescent with treatment-resistant schizophrenia?

Treatment-resistant schizophrenia is defined by persistence of significant symptoms, despite adequate treatment with at least two different antipsychotics, lasting 6 weeks each, and during which there was no appreciable symptomatic or functional improvement, as measured by validated rating instruments (Howes et al., 2017). It can be present from the beginning of therapy or develop over time, often after relapses.

Before considering that a patient has a treatment-resistant schizophrenia, we need to investigate possible causes of lack of effectiveness of prescribed treatments (Abidi et al., 2017; NICE, 2013). After reviewing diagnosis, we should document adequate treatment adherence and make sure that optimal medication dosages have been used. In children and adolescents, antipsychotics should not be dosed according to weight, but rather using a ‘start slow, go slow’ approach, raising the dose until reaching recommended maximum doses or until adverse events appear. If a trial cannot be completed at the adequate dose, then another treatment should be tried until two adequate trials of different antipsychotics are completed. Adequate treatment of medical and psychiatric comorbidities should also be implemented (medication or psychosocial interventions as needed), paying attention to potential drug interactions that may increase adverse effects or diminish efficacy of antipsychotic medication.

According to NICE guidelines, clozapine should be offered to children and young people with treatment-resistant schizophrenia (NICE, 2013). This recommendation is based on evidence of clozapine being significantly more effective than other antipsychotics in children and adolescents (Krause et al., 2018). Clozapine monitoring trough plasma levels may help to guide dosing. Greatest efficacy is seen at levels ⩾350 µg/L, although this level is not universal, and factors such as gender, inflammation or tobacco or caffeine consumption may alter clozapine plasma levels (Correll and Howes, 2021).

Initiation of clozapine requires a careful balance of the risk/benefit ratio and commitment to the increased monitoring requirements by patient, family and psychiatrist. Psychiatrists should adopt a proactive approach on assessment, intervention and reassurance for patients and families, as most side effects will appear during the first weeks of treatment and could be managed without discontinuation (Correll and Howes, 2021).

### How do I manage weight increase related to the use of antipsychotic medications in children and adolescents?

Antipsychotics are associated with weight gain and adverse metabolic effects, particularly in children and adolescents, although mechanisms driving these effects are still largely unknown (Libowitz and Nurmi, 2021). However, not all antipsychotics carry the same risk. In randomised clinical trials clozapine, olanzapine and quetiapine showed the highest weight gain, while molindone, lurasidone and ziprasidone (which was not more efficacious than placebo) induced less weight gain (Krause et al., 2018). In observational studies, olanzapine and clozapine displayed the highest risk of weight gain, followed by risperidone, quetiapine and aripiprazole, and ziprasidone was associated with no weight gain (Pozzi et al., 2022). Longer time in treatment and being drug-naïve also increased risk, with a ceiling effect determined by higher baseline BMI values (Pozzi et al., 2022). Therefore, as there are no differences in efficacy (except for clozapine and ziprasidone), choosing an antipsychotic with less potential for weight increase, particularly if this is the first antipsychotic trial, together with education about strategies to enhance appropriate nutritional style and physical activity, and regular weight monitoring (Barlow, 2007) may help to limit weight gain.

In case weight gain is established, treatment options could include switching to a less orexigenic/metabolically adverse antipsychotic, adjunctive behavioural treatments and adjunctive pharmacologic interventions. The IMPACT trial (Correll et al., 2020), enrolled obese/overweight youth 8–19 years with psychotic disorders and with weight gain after treatment with antipsychotics, and randomised them to adjunctive metformin, antipsychotic switch or control intervention, with all arms receiving healthy lifestyle education. Both active interventions were significantly different compared to the control condition, with no differences between them, but with more gastrointestinal problems in the metformin group. However, weight reduction with aripiprazole and metformin was modest, highlighting the importance of considering potential risks and benefits prior to antipsychotic initiation. Switching strategy to newly FDA approved agents with better weight and metabolic profile such as lurasidone – a full antagonist at dopamine D2 and 5-HT2A and 5-HT7 receptors, or brexpiprazole – a D2 and D3, as well as 5-HT1A receptor partial agonist could be an option, although data supporting this option are still not available.

## Tics/Tourette syndrome

### Is it best to replace methylphenidate with guanfacine or use it as adjunct when ADHD is present but tics require treatment as well?

As both methylphenidate and guanfacine are licensed for the treatment of ADHD, both can be helpful and effective treatments for this condition; however, the co-occurrence of tics is unlikely to be treated adequately using methylphenidate. While treating ADHD with methylphenidate can have beneficial effects on associated symptoms that increase tics (frustration, impulsivity, emotional dysregulation, etc.), there does not appear to be a direct effect on tics.

The clinical decision whether to switch to guanfacine or to augment methylphenidate with guanfacine is based on: (i) the relative severity of ADHD and tic symptoms, (ii) whether methylphenidate is effectively managing the ADHD symptoms, and (iii) whether methylphenidate appears to be worsening tics. If methylphenidate is already effective in treating ADHD symptoms, but tics worsen and/or require treatment, guanfacine may be added. In cases of mild to moderate ADHD symptoms with co-existing tics, and/or if methylphenidate is only partially effective or poorly tolerated, discontinuation of methylphenidate and switching to guanfacine may be considered. If tics improve with guanfacine alone but ADHD worsens, then methylphenidate may be restarted. In the cases of mild-to-moderate ADHD with co-existing tics (where both require treatment) there is an argument to suggest that guanfacine monotherapy should be considered as the first-line medication choice rather than methylphenidate. However, there remains clinical uncertainty about the best treatment choice in these circumstances, and in the UK the current HTA SATURN trial (https://fundingawards.nihr.ac.uk/award/NIHR128472) is examining this question in a head-to-head comparison of guanfacine versus methylphenidate for ADHD.

In case of moderate-to-severe ADHD with tics, ADHD symptoms are likely to require treatment with psychostimulants which are the first-line treatment choice for ADHD. Therefore, in cases such as this where tics are also present, augmenting methylphenidate with guanfacine to cover both the symptoms of both conditions seems preferable. Using guanfacine in this respect also may benefit ADHD symptoms as shown by McCracken et al. (2016). Adverse events were generally mild to moderate, and combined treatment showed no differences in safety or tolerability.

While appreciating that when prescribing for children and young people it is best for children to receive as few medications as possible at the lowest dose to achieve the desired clinical goals, when there is clear impairment from both disorders, it may be beneficial to combine treatment. Therefore, especially when ADHD symptoms are more severe and impairing, a combination of methylphenidate and guanfacine is worth considering.

The addition of guanfacine can also potentially benefit sleep difficulties and night-time settling, and decrease the need for alternatives such as melatonin which may be beneficial to consider.

### When would I consider antipsychotics more appropriate than clonidine/guanfacine when treating tics in children and young people?

In a recent prescribing survey among 59 European expert clinicians who were members of the European Society for the Study of Tourette Syndrome (Roessner et al., 2022) the most commonly used medications for tics in children and adolescents in descending order were aripiprazole, clonidine, tiapride (not available in the United Kingdom) and guanfacine. Newer (so called) antipsychotics (e.g. risperidone and aripiprazole) and noradrenergic agents (e.g. clonidine and guanfacine) have increasingly been favoured over the older so-called antipsychotic drugs (e.g. pimozide, sulpiride and haloperidol, all D2 receptor antagonists). Although a recent systematic review and meta-analysis shows that the efficacy for tic treatment appears similar between noradrenergic and antipsychotic medications (Hollis et al., 2016; Whittington et al., 2016), the Maudsley prescribing guidelines (Taylor et al., 2021) state that because of the more serious adverse effects of antipsychotics, it is recommended that noradrenergic drugs (i.e. clonidine and guanfacine) are used first-line. The Maudsley guidelines go on to however recognise that the antipsychotic medications may be more beneficial in some individuals, although there is no clear guidance as to which group of children this might pertain to.

One possible advantage of using antipsychotic medications over other options is that medication such as risperidone or aripiprazole could have a more favourable effect on some of the behavioural symptoms associated with tic disorders and on the common comorbidities such as ASD and OCD. Risperidone, particularly, has proven efficacy in ameliorating aggressive behaviour (Sandor and Stephens, 2000) and therefore in the case of challenging behaviour either linked to autism spectrum disorder or ADHD, risperidone and aripiprazole are likely to have more significant effects on aggression than noradrenergic counterparts. It is worth noting though that noradrenergic options such as clonidine or guanfacine are likely to have much more of an effect on ADHD core symptoms, and noradrenergic drugs are recommended as the first-line option for tic disorders with co-morbid ADHD in the recent European Guidelines (Roessner et al., 2022).

Risperidone also has a greater affinity for 5-HT receptors and is more likely to augment serotonergic agents in the treatment of obsessive-compulsive symptoms (OCSs). Therefore, when a combined Tic and OCS presentation is present and obsessive and/or compulsive symptoms are equally problematic, antipsychotics have often been used alongside SSRI’s. This effect has also been shown to be present with aripiprazole, and the partial agonist profile of this medication may point to it being more effective on mood and OCS difficulties. One of the potential side effects of noradrenergic agents is depression (Joint Formulary Committee, 2022).

Typically, antipsychotic medications are also more sedating than noradrenergic alternatives. This might, for example, be beneficial to consider in young people who already using sedating medications such as promethazine.

Of note, noradrenergic agents are contraindicated in individuals who experience severe bradyarrhythmia secondary to second- or third-degree AV block or sick sinus syndrome. Antipsychotic medications are not contraindicated in this type of patients, although cardiology opinion would be essential before prescribing antipsychotic medication in this client group. Aripiprazole has the least cardiac side effect profile of the antipsychotic medications especially related to QT prolongation.

### When clonidine is being used in higher doses is it best to go beyond treatment window or consider a switch to guanfacine? If switching, is it best to reduce and withdraw clonidine before initiating guanfacine or can you cross-taper?

Recommended clonidine therapeutic doses are in the order of 3–5 mcg/kg/day. It is uncommon to use doses beyond 300 mcg daily, due to the sedating and hypotensive effects of clonidine. However, some young people may be able to tolerate the cardiac effects of clonidine well and find that the sedation has additional benefits to sleep and comorbidities. Therefore, in these children, higher doses may be beneficial with regular monitoring of blood pressure and pulse. As clonidine can also be divided into two or three doses, quite often children who are sensitive to guanfacine side effects find that a BD or TDS regime of clonidine suits them better than the once daily dosing of guanfacine.

Clonidine is 10 times more potent than guanfacine at alpha-2 presynaptic receptors, whereas guanfacine appears to be more potent at post synaptic receptors, which translates into increased prefrontal activity and impulse control regulation and improvement in behaviour regulation (Cinnamon Bidwell et al., 2010). Therefore, in the case of comorbidities where behavioural modification is desired, guanfacine seems to have a better effect.

Guanfacine weight dosing guidelines indicate 0.05–0.08 mg/kg/day suggesting that a rough equivalency is: Guanfacine 1 mg = Clonidine 100 mcg.

If it is clinically acceptable from a tic perspective to reduce and withdrawal clonidine first, before initiating guanfacine, this is likely to be best practise, as the side effects will be additive. Reducing clonidine by 25 mg every 3–5 days is thought to be appropriate to facilitate this reduction.

However, it may not be beneficial from a tic/sleep/behaviour perspective to reduce and withdraw clonidine completely before starting guanfacine. In these circumstances, cross-tapering is recommended (Elbe, 2020) by reducing clonidine to 100 mcg daily before initiating guanfacine at 1mg daily. Following on from this, all remaining clonidine doses are reduced by 25 mcg every 3 days before guanfacine is increased as necessary.

## Conclusions

We hope that this article will be helpful for prescribers in their daily clinical practice. We also hope that in the near future, additional high-quality evidence, applicable at the individual patient, rather than group level, will inform the answers to these and other important questions, within the framework of a precision psychiatry approach (Cortese, 2021).
